# Characterizing T cell responses to enzymatically modified beta cell neo-epitopes

**DOI:** 10.3389/fimmu.2022.1015855

**Published:** 2023-01-10

**Authors:** Hai Nguyen, David Arribas-Layton, I-Ting Chow, Cate Speake, William W. Kwok, Martin J. Hessner, Carla J. Greenbaum, Eddie A. James

**Affiliations:** ^1^ Center for Translational Immunology, Benaroya Research Institute at Virginia Mason, Seattle, WA, United States; ^2^ Department of Pediatrics, The Medical College of Wisconsin, Milwaukee, WI, United States; ^3^ Department of Medicine, University of Washington, Seattle, WA, United States

**Keywords:** T cell, citrullinated autoantigens, deamidated autoantigens, Type 1 diabetes (T1D), at risk

## Abstract

**Introduction:**

Previous studies verify the formation of enzymatically post-translationally modified (PTM) self-peptides and their preferred recognition by T cells in subjects with type 1 diabetes (T1D). However, questions remain about the relative prevalence of T cells that recognize PTM self-peptides derived from different antigens, their functional phenotypes, and whether their presence correlates with a specific disease endotype.

**Methods:**

To address this question, we identified a cohort of subjects with T1D who had diverse levels of residual beta cell function. Using previously developed HLA class II tetramer reagents, we enumerated T cells that recognize PTM GAD epitopes in the context of DRB1*04:01 or PTM IA2 epitopes in the context of DQB1*03:02 (DQ8).

**Results:**

Consistent with prior studies, we observed higher overall frequencies and a greater proportion of memory T cells in subjects with T1D than in HLA matched controls. There were significantly higher numbers of GAD specific T cells than IA2 specific T cells in subjects with T1D. T cells specific for both groups of epitopes could be expanded from the peripheral blood of subjects with established T1D and at-risk subjects. Expanded neo-epitope specific T cells primarily produced interferon gamma in both groups, but a greater proportion of T cells were interferon gamma positive in subjects with T1D, including some poly-functional cells that also produced IL-4. Based on direct surface phenotyping, neo-epitope specific T cells exhibited diverse combinations of chemokine receptors. However, the largest proportion had markers associated with a Th1-like phenotype. Notably, DQ8 restricted responses to PTM IA2 were over-represented in subjects with lower residual beta cell function. Neo-epitope specific T cells were present in at-risk subjects, and those with multiple autoantibodies have higher interferon gamma to IL-4 ratios than those with single autoantibodies, suggesting a shift in polarization during progression.

**Discussion:**

These results reinforce the relevance of PTM neo-epitopes in human disease and suggest that distinct responses to neo-antigens promote a more rapid decline in beta cell function.

During the development of type 1 diabetes (T1D), pancreatic β-cells are selectively destroyed, eventually leading to dependence on exogenous insulin. The dominant genetic association of T1D with HLA class II genes (1) and observed enrichment of autoimmune associated single nucleotide polymorphisms within CD4 T cell superenhancers (2) provide evidence that CD4 T cell responses play an important role in the disease. Indeed, I-Ag7-restricted responses against insulin B 9-23 play a crucial role in the NOD diabetes model (3) and CD4+ T cell responses toward multiple β-cell proteins have been observed in human T1D (4). T cell receptors (TCR) with inappropriate recognition of self-peptides are thought to undergo negative selection or diversion to a regulatory lineage (5, 6). However, healthy individuals with HLA haplotypes that predispose to autoimmunity have been shown to have a potentially autoreactive T cell repertoire, indicating that negative selection is imperfect (7). Immune escape can be facilitated by the formation of neo-epitopes, which may be under-represented in the thymus but can be recognized with higher affinity in the periphery due to enhanced HLA binding and/or TCR recognition (8). Among several classes of post-translationally modified (PTM) epitopes, specific recognition of citrullinated and deamidated neo-epitopes derived from beta cell antigens has been directly demonstrated (9–12). Further underscoring their potential relevance, responses to some of these PTM epitopes were documented among the islet-infiltrating T cells of pancreatic organ donors (12, 13).

We previously developed HLA class II tetramers that facilitate direct enumeration and study of T cells that recognize PTM glutamic acid decarboxylase 65 (GAD) epitopes in the context of DRB1*04:01 and PTM insulinoma-associated protein-2 (IA2) epitopes in the context of DQB1*03:02 (4, 10). Applying those tetramers in separate cohorts of subjects, we observed that T cells that recognize these PTM neo-epitopes were present at increased frequencies in subjects with T1D and limited data suggested a Th1 phenotype for these T cells (10, 12). However, important questions remain about relative prevalence of T cells that recognize these two distinct PTM antigens and whether their presence or functional phenotypes correlate with a specific disease endotype. Notably, residual beta-cell function (effectively estimated by measuring levels of serum c-peptide) is present at diverse levels in subjects previously diagnosed with T1D (14, 15). Those who can produce small but clinically significant amounts of insulin have been shown to have lower glycated hemoglobin A1c levels, less hypoglycemia, and a reduced incidence of long-term complications (16, 17), suggesting that subjects with comparatively high or low residual c-peptide may represent distinct disease endotypes. In light of this, the current study was designed to probe the attributes of PTM epitope specific CD4+ T cells in a cross-sectional cohort of subjects with T1D with either high or low levels of residual c-peptide and in at-risk subjects with single or multiple autoantibodies. This was accomplished by investigating the frequency of PTM GAD and PTM IA2 specific T cells through direct tetramer staining and by assessing their cytokine profiles and surface phenotype. Our results indicated that subjects with T1D who had either high or low levels of residual beta cell function exhibited different frequency distributions of PTM specific T cells. Furthermore, PTM specific T cells exhibited different functional profiles in at-risk subjects with single versus multiple autoantibodies.

## Research design and methods

### Human subjects

A total of 25 subjects with T1D with HLA-DRB1*04:01 (DR0401) and DQB1*03:02 (DQ8) haplotypes were recruited for the study, including 19 recruited through the T1D Exchange Biobank and 6 recruited through the Benaroya Research Institute Diabetes Registry, all with informed consent and under approved protocols (**Supplemental Table 1**). At the time of recruitment, subjects recruited through the T1D Exchange were characterized for serum islet autoantibodies and their peak C-peptide (a measure of residual beta cell function) was determined using a mixed meal tolerance test. The subjects had diverse peak levels of c-peptide that could be conveniently divided into groups with high (>0.2 nmol/L) or low (<0.2 nmol/L) levels of residual beta cell function, but were otherwise well matched (**Supplemental Figure 1**). A total of 16 HLA matched healthy control subjects and no family history of autoimmunity (**Supplemental Table 2**) were recruited though the Benaroya Research Institute (BRI) control registries with informed consent under protocols approved by their Institutional Review Board. The healthy subjects had a significantly higher age at draw, but were well matched with respect to biological sex (**Supplemental Figure 2**). Autoantibody positive at risk subjects were recruited through the diabetes clinic at Children’s Wisconsin with parental consent and informed assent under a protocol (CHW IRB 01–15) approved by their Institutional Review Board (**Supplemental Table 3**).

### HLA class II tetramer reagents

DRB1*04:01 (DR0401) and DQB1*03:02 (DQ8) protein was purified from insect cell cultures as previously described (18, 19). Briefly, DR0401 or DR0401-myc, and DQ0302 protein was purified from supernatants by affinity chromatography, biotinylated with biotin ligase, and dialyzed against phosphate buffer (0.0625 M monobasic sodium phosphate, 0.0375 M monobasic sodium phosphate, pH 6.0). To prepare multimers DR0401, DR0401-myc, or DQ8 monomer was incubated with 0.2 mg/mL peptide, 0.2% n-octyl-β-d-glucopyranoside (Sigma) and 1 mM Pefabloc SC (Sigma) at 37 °C for 72 h. After incubation PE labeled streptavidin (ThermoFisher), PE-CF594 labeled streptavidin (BD), or BV421 labeled streptavidin (BD) was added to conjugate monomers into tetramers. As summarized in **Table 1**, DR0401 restricted epitopes were labeled with PE, whereas DQ8 restricted epitopes were labeled with PE-CF594. The viral control epitope, influenza matrix protein (MP) 97–116, was prepared with myc tagged DR0401 and labeled with BV421.

**Table 1:** Summary of PTM epitope tetramers used for T cell studies.

Peptide	Modification	Amino acid sequence^a,b^	HLA	Label
GAD 114-128	120-E	VMNILL **E** YVVKSFDR	DR0401	PE
GAD 153-172	154-E, 164-E	P**E**NLEEILMHC **E** TTLKYAIK	DR0401	PE
GAD 265-284	272-Cit	KGMAALP **X** LIAFTSEHSHFS	DR0401	PE
IA2 198-216	207-E, 213E	SLSYEPALL **E** PYLFH **E** FGS	DQ8	PE-CF594
IA2 467-482	478-E	AAEEYGYIVTD **E** KPLS	DQ8	PE-CF594
IA2 523-536	532-E, 533-E	QNLSLADVT **EE** AGL	DQ8	PE-CF594
IA2 545-562	548-E, 551-E, 556-E	TGL**E**IL **E** TGVG **E** REEAAA	DQ8	PE-CF594
MP 97-116	(none)	VKLYRKLKREITFHGAKEIS	DR0401	BV421

a PTM residues are bolded in each sequence (E indicates a deamidated glutamine residue, X indicates a citrullinated arginine residue).

b The predicted minimal epitope is underlined.

c DR0401-restricted PTM epitopes, DQ8-restricted PTM epitopes, and the reference influenza epitopes were assigned unique fluorescent labels to allow grouped analysis.

### Ex vivo tetramer analysis

Analysis of T cell frequency was accomplished using our previously published approach (20). Briefly, 20-30×10^6^ PBMCs were thawed and washed in the presence of Benzonase (1:5000 dilution), and re-suspended in a total of 200-400 µL of T cell media (standard RPMI supplemented with 10% pooled human serum, 1% penicillin-streptomycin, 1% l-glutamine). After diverting 4 × 10^6^ cells to set up *in vitro* culture (see next section), the remaining PBMC were subjected to direct tetramer staining. Individual preparations of each single tetramer (listed in **Table 1**) were prepared at a final concentration of 0.5 mg/mL as described above. A master mix of multimers was then prepared by combining 5 uL of each tetramer. PBMCs were stained with the multimer master mix for 120 min at room temperature in the dark, washed, and then incubated with anti-PE and anti-Myc magnetic beads (Miltenyi Biotec) for 20 min at 4 °C. Cells were washed twice and 2.5% of the cells were saved for analysis as a “pre-enrichment” sample. The remaining cells were enriched with a magnetic column, then removed from column, flushed and collected. Enriched and pre-column samples were stained with CD4-BV650 (BD Biosciences, Clone RPA-T4), CD45RA-AF700 (BioLegend, Clone HI100), CXCR3-FITC (BioLegend, Clone G025H7), CCR4-BV605 (BioLegend, Clone L291H4), CCR6-BV510(BD Biosciences, Clone 11A9) and a combination of CD14-PerCP-Cy5.5 (BioLegend, Clone M5E2), and CD19-PerCP-Cy5.5 (BioLegend, Clone 6D5) for 15 min at 4 °C. Samples were washed then labeled with ViaProbe (BD Biosciences) and run on a BD LSRII. Frequencies were calculated as previously described (20). Live tetramer positive T-cells were sequentially gated (see **Supplemental Figure 3**) and then classified as naïve or memory based on CD45RA expression (a minimum number of 5 tetramer positive events was required). To assign T cell lineages, memory T cells subdivided into Th1 (CXCR3+, CCR4- and CCR6-), Th2 (CXCR3-, CCR4+ and CCR6-), Th17 (CXCR3-, CCR4+ and CCR6+), Th1/17 (CXCR3+, CCR4- and CCR6+), Th1/2 (CXCR3+, CCR4+ and CCR6-), or Th1* (CXCR3+, CCR4+ and CCR6+), CCR6 only (CXCR3-, CCR4- and CCR6+), or CR neg (CXCR3-, CCR4- and CCR6-) lineages (a minimum number of 10 tetramer positive events was required).

### *In vitro* T cell expansion

Thawed PBMC were plated at 4 × 10^6^ cells/mL in T cell media (standard RPMI supplemented with 10% pooled human serum, 1% penicillin-streptomycin, 1% l-glutamine), and stimulated with a pool of beta cell derived peptides (see **Table 1**) in 48 well plates at a total concentration of 20 µg/mL. Cultures were supplemented with IL-2 starting after 7 days and split into new wells as needed. After 14 days, *in vitro* expanded T cells were stained with the corresponding DR0401 and DQ8 tetramers at 37 °C for 60 min and then stained with CD25 FITC (BioLegend, Clone PC61), CD3 APC (BioLegend, Clone UCHT1), and CD4 PerCP (BioLegend, Clone OKT4) at 4 °C for 15 min. Samples were analyzed on a BD LSRII and FlowJo and wells with expanded tetramer positive T cells were analyzed by intracellular cytokine staining.

### Intracellular cytokine staining of epitope specific T cells

Expanded T cell cultures were rested for 3 days, resuspended in 200 µl of T cell medium, stained with 2.5 uL of each appropriate tetramer, and incubated at 37°C for 30 min, and then activated with 50 ng/mL phorbol 12-myristate 13-acetate and 1 µg/mL ionomycin in the presence of 10 µg/mL Brefeldin A for 4 hours at 37°C. After activation, cells were stained with CD3 PE-Cy5 (clone HIT3a, BioLegend) and CD4 v500 (clone RPA-T4, BD Biosciences) as well as Fixable Viability Stain 450 (BD Horizon). Cells were then fixed and permeabilized as per the manufacturer’s instructions (eBioscience). Permeabilized cells were then stained with antibodies against IFN-γ AF700 (clone 4S.B3, BioLegend), IL-10 APC H7A (clone MQ1-17H12, BioLegend), IL-17A PE Cy7 (clone BL168, BioLegend), and IL-4 FITC (clone 8D4-8, eBioscience) for 20 minutes at 4°C. Cells were then washed in PBS and immediately analyzed by flow cytometry on a BD LSRII multi-color flow cytometer and analyzed using FlowJo (Treestar Inc). Clones were considered cytokine positive if more than 10% of the cells produced that particular cytokine.

### Statistics

All statistical tests were performed using PRISM software (GraphPad) or the VassarStats online contingency analysis tools (http://vassarstats.net/index.html). A Mann Whitney test was used for two group comparisons between T1D subjects and controls or between single versus multiple antibody positive at-risk subjects. A Wilcoxon matched-pairs signed rank test was used to compare GAD and IA2 specific frequencies. Simple linear regression was applied to probe correlations between levels of residual c-peptide and T cell frequencies.

## Results

### T cells that recognize neo-epitopes are present at elevated frequencies in subjects with T1D

**Figure 1:**
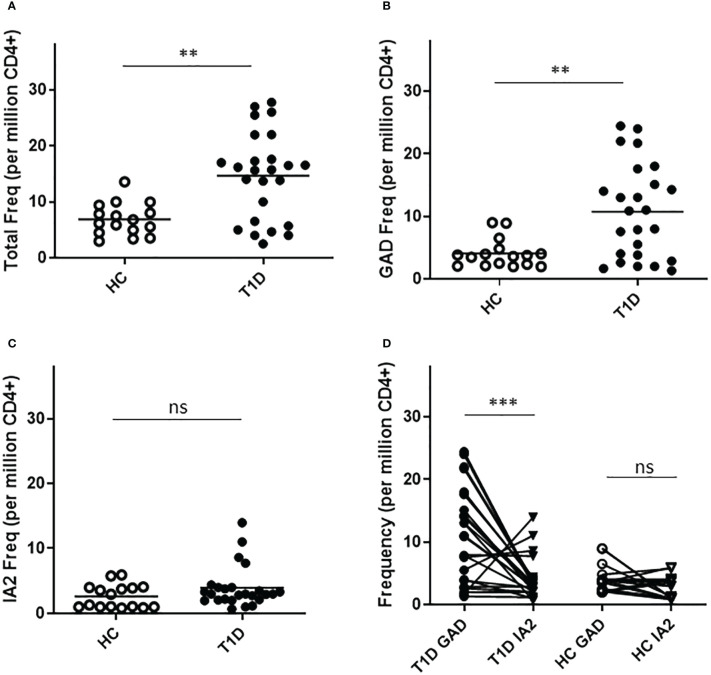
T cells specific for PTM epitopes are more frequent in subjects with T1D. HLA class II tetramers were used to characterize T cells specific for PTM GAD (DR0401 restricted) and PTM IA-2 (DQ8 restricted) epitopes in 25 subjects with T1D and 15 HLA matched control subjects. **(A)** Combining both groups of epitopes, total frequencies were significantly higher (p=0.0017, Mann Whitney test) in subjects with type 1 diabetes (black circles) than in HLA matched controls (white circles). **(B)** Examining PTM GAD and IA-2 epitopes separately, frequencies were significantly higher for PTM GAD (p=0.0069, Mann Whitney test) in subjects with type 1 diabetes (black circles) than in healthy controls (white circles). **(C)** In contrast, although four subjects had elevated frequencies, The frequency of IA-2 specific T cells was not significantly higher (p=0.33, Mann Whitney test) in subjects with type 1 diabetes (black circles) than in healthy controls (white circles). **(D)** Performing a paired comparison of PTM GAD and PTM IA2 specific T cell frequencies, PTM GAD frequencies were significantly higher than PTM IA2 frequencies in subjects with T1D (p=0.0004, Wilcoxon matched-pairs signed rank test), but were not significantly different in matched controls (p=0.22, Wilcoxon matched-pairs signed rank test). Horizontal lines in each panel indicate the mean value for each group. ns indicates "not significant" (p-value >0.05) * indicates p-value below 0.05 but above 0.01 ** indicates p-value below 0.01 but above 0.001 *** indicates p-value below 0.001 but above 0.0001.

To confirm the relevance of T cells that recognize neo-epitopes from GAD and IA2, we applied a direct tetramer enrichment approach (20) to measure their frequency in the peripheral blood of subjects with T1D. To assess the relative frequencies of PTM epitope reactive T cells, we labeled a group of DR0401 restricted PTM GAD epitopes (PTM GAD) with PE-labeled tetramers and a group of DQ8 restricted PTM IA2 epitopes (PTM IA2) with PE-CF594-labeled tetramers, as summarized in **Table 1**. The total frequency of PTM epitope reactive T cells (PTM GAD and PTM IA2 combined) was significantly higher in subjects with T1D than in HLA matched controls (p=0.0017, **Figure 1A**). Comparing the frequencies of PTM GAD epitopes and PTM IA2 epitopes separately, the frequency of PTM GAD reactive T cells was significantly higher in subjects with T1D than in HLA matched controls (p=0.0069, **Figure 1B**) whereas the frequency of PTM IA2 reactive T cells was elevated in a few subjects but not significantly higher in subjects with T1D than in HLA matched controls (p=0.33, **Figure 1C**). A paired analysis revealed that most subjects with T1D had significantly higher frequencies of PTM GAD specific T cells as compared with IA2 specific T cells (p=0.0004, **Figure 1D**), whereas PTM GAD and IA2 specific frequencies did not differ in controls (p=0.22, **Figure 1D**).

### T cells specific for PTMs predominantly exhibit a Th1-like functional profile

**Figure 2:**
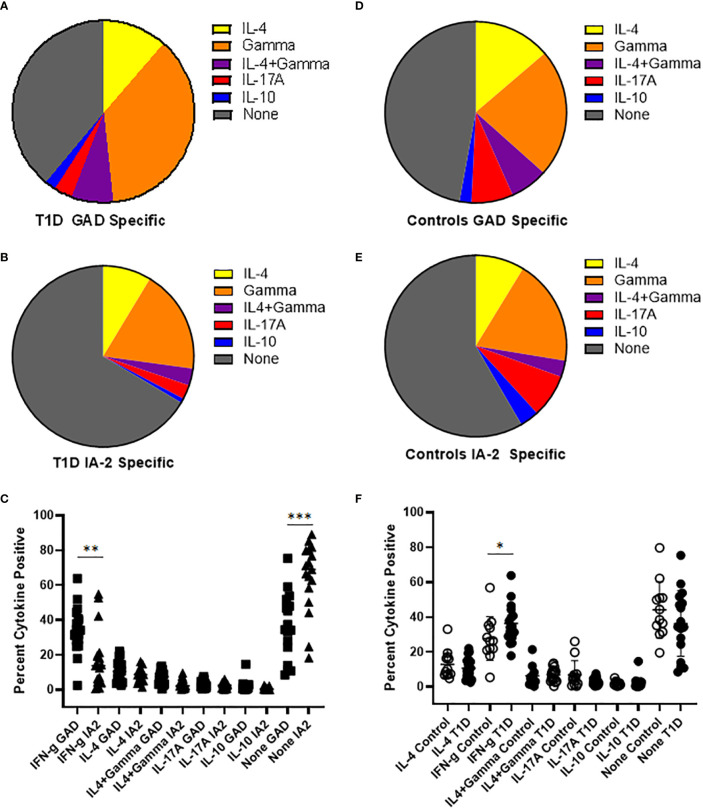
PTM GAD epitope specific T cells have a distinct cytokine profile in subjects with T1D. PTM GAD and IA-2 epitope specific T cells were characterized by intracellular cytokine staining after a single round of *in vitro* expansion. **(A)** The cytokine profiles of PTM GAD specific T cells were examined for 17 subjects with T1D. Taking the average across all subjects, cells that produced interferon-γ only were predominant (34.4%), followed by IL-4 only (10.7%), IL4+ interferon-γ (6.9%), IL-17A (3.0%), and IL-10 (1.9%); a notable proportion were cytokine negative (36%). **(B)** The cytokine profiles of PTM IA-2 specific T cells were examined in the same 17 subjects with T1D. Taking the average across all subjects, cells that produced interferon-γ only were predominant (18%) followed by IL-4 only (8.4%), IL4+ interferon-γ (2.9%), IL-17A (2.5%), and IL-10 (0.8%); a major proportion were cytokine negative (65%). **(C)** Comparing PTM GAD (square symbols) and PTM IA-2 specific T cells (triangles) in subjects with T1D, a significantly higher percentage of PTM GAD specific T cells produced interferon-γ only (p=0.0033, Mann Whitney test) and a significantly lower percentage were cytokine negative (p=0.0003). **(D)** The cytokine profiles of PTM GAD specific T cells were also examined in 12 control subjects. Taking the average across all subjects, cells that produced interferon-γ only were predominant (21.4%) followed by IL-4 only (12.9%), IL-17A (7.0%), IL4+ interferon-γ (6.3%), and IL-10 (2.0%); a major proportion were cytokine negative (44%). **(E)** The cytokine profiles of PTM IA-2 specific T cells were examined in the same 12 control subjects. Taking the average across all subjects, cells that produced interferon-γ only were predominant (18.3%) followed by IL-4 only (8.5%), IL-17A (7.5%), IL-10 (3.3%), and IL4+ interferon-γ (2.9%); a major proportion were cytokine negative (57%). **(F)** Comparing subjects with T1D (filled circles) and controls (open circles), a significantly higher percentage of PTM GAD specific T cells produced interferon-γ only (p=0.05, Mann Whitney test). No other difference was significant. Horizontal lines in each panel indicate the mean value for each group. Vertical lines indicate standard deviations. * indicates p-value below 0.05 but above 0.01 ** indicates p-value below 0.01 but above 0.001 *** indicates p-value below 0.001 but above 0.0001.

To draw inferences about the functional phenotypes of T cells that recognize PTM epitopes, we performed cytokine profiling and surface phenotyping of tetramer positive T cells. For cytokine profiling, it was necessary to perform a single round of *in vitro* expansion, followed by intracellular cytokine staining. T cells specific for PTM GAD or IA2 were successfully expanded (in separate wells, using the peptides summarized in **Table 1**) for peripheral blood samples from 17 subjects with T1D and 12 controls (for the remaining subjects cultures failed to expand, most likely for technical reasons). We then rested the cells and performed intracellular cytokine staining analysis of tetramer positive CD4+ T cells. In subjects with T1D, most of the expanded epitope specific T cells were positive for a single cytokine, but a non-negligible fraction co-produced interferon-γ and IL-4. Interferon-γ was the predominant cytokine for both PTM GAD (**Figure 2A**) and PTM IA2 (**Figure 2B**), followed by IL-4, IL-17A, and IL-10. However, detectable levels of cytokine were comparatively more robust for PTM GAD than for PTM IA2 (**Figure 2C**), exhibiting significantly higher proportions of epitope specific T cells that were produced interferon-γ only (p=0.0033) and a significantly lower percentage that were cytokine negative (p=0.0003). The cytokine profiles of PTM GAD (**Figure 2D**) and PTM IA2 (**Figure 2E**) specific T cells exhibited a similar cytokine hierarchy in control subjects (interferon-γ > IL-4 > IL-17A > IL-10), but there were no significant differences between the cytokine levels for PTM GAD and PTM IA2 (**Supplemental Figure 4A**). Comparing subjects with T1D and controls, a significantly higher percentage (p=0.008) of PTM GAD specific T cells produced interferon-γ only (**Figure 2F**). In contrast, no significant differences were observed for PTM IA2 specific T cells (**Supplemental Figure 4B**).

**Figure 3:**
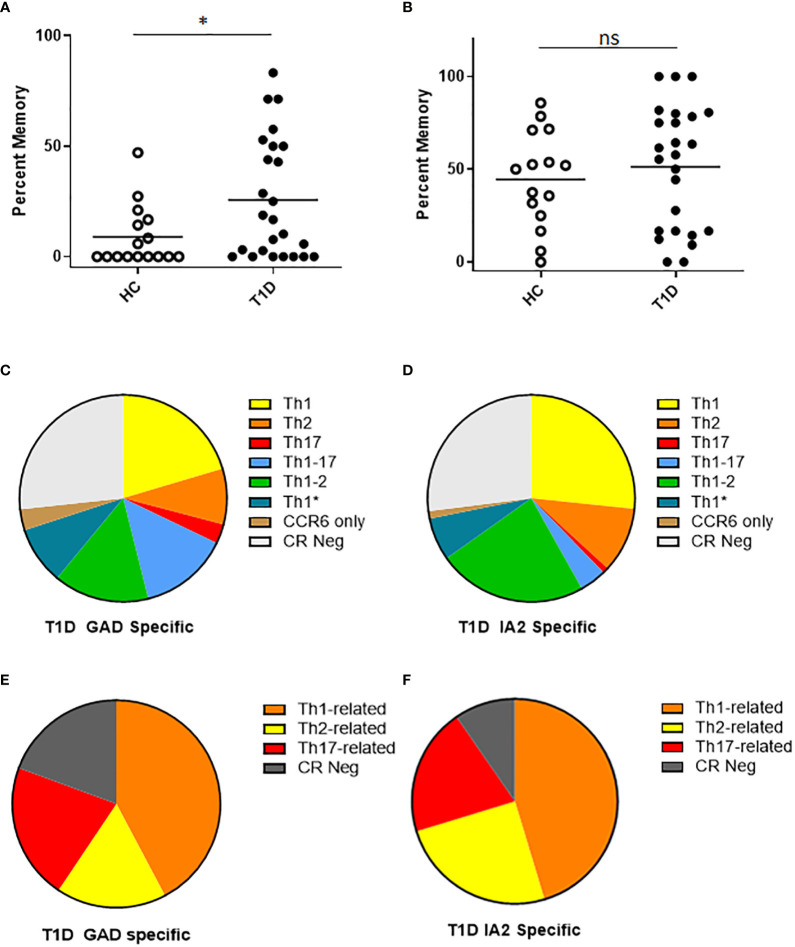
T cells specific for PTM GAD epitopes have distinct memory phenotype in subjects with T1D. We characterized the surface phenotype of T cells specific for PTM GAD (DR0401 restricted) and PTM IA-2 (DQ8 restricted) epitopes in 25 subjects with T1D and 15 HLA matched control subjects. **(A)** Defining CD45RA negative cells as conventional memory T cells, the percentage of PTM GAD specific T cells that had a memory phenotype was significantly higher (p=0.0368, Mann Whitney test) in subjects with type 1 diabetes (black circles) than in healthy controls (white circles). **(B)** In contrast, the percentage of IA-2 specific T cells that had a memory phenotype did not differ (p=0.47, Mann Whitney test) between subjects with type 1 diabetes (black circles) and healthy controls (white circles). **(C)** Using combinations of chemokine receptors, the lineage distribution PTM GAD specific memory T cells was examined for subjects with T1D. Taking the average across all subjects, Th1-like cells were predominant (20.3%) followed by Th1-2 (14.9%), Th1-17 (14.0%), Th1* (8.9%), Th2 (8.7%), and Th17 (3.0%); a notable proportion were chemokine receptor negative (27%) and a minor proportion were positive for CCR6 only (3.3%). **(D)** In the same manner, the lineage distribution of PTM IA2 specific memory T cells was examined for subjects with T1D. Taking the average across all subjects, Th1-like cells were predominant (26.6%) followed by Th1-2 (23.3%), Th2 (10.2%), Th1* (6.7%), Th1-17 (4.2%), and Th17 (0.9%); a notable proportion were chemokine receptor negative (27.0%) and a minor proportion were positive for CCR6 only (1.2%). **(E)** To juxtapose functional cytokine and surface marker data, we combined observed proportions of interferon-γ-associated (Th1, Th1-2, Th1-17, and Th1*) IL-4-associated (Th2 and Th1-2), and IL-17-associated (Th17, Th1-17, Th1*, and CCR6 only) lineage combinations for PTM GAD specific T cells. Taking the average across all subjects, interferon-γ-associated cells were predominant (42.2%) followed by IL-17-associated (21.1%), chemokine receptor negative (19.3%), and IL-4-associated (17.1%) cells. **(F)** In the same manner we combined observed proportions of interferon-γ-associated, IL-4-associated, and IL-17-associated PTM IA2 specific memory T cells. Taking the average across all subjects, interferon-γ-associated cells were predominant (45.3%) followed by IL-4-associated (24.9%), chemokine receptor negative (20.1%), and IL-17-associated (9.6%) cells. Horizontal lines in each panel indicate the mean value for each group. not significant (p-value > 0.05).

For surface phenotyping, in concert with direct HLA class II tetramer staining, T cells were co-stained with antibodies specific for surface markers to draw inference about the memory status and lineages of PTM epitope specific T cells. Here, we first focused on CD54RA negative cells to compare the relative proportion of conventional memory T cells. We observed that subjects with T1D had a significantly higher percentage of PTM GAD specific T cells that had a memory phenotype (**Figure 3A**), whereas there was no significant difference in the percentage of PTM IA2 specific T cells that had a memory phenotype (**Figure 3B**). Frequencies were comparatively higher in subjects with T1D, allowing a further granular analysis of surface phenotype that could not be done for control subjects. For this we excluded naïve (CD45RA+CCR7-) cells and pooled the remaining memory T cells and subdivided them into Th1 (CXCR3+, CCR4- and CCR6-), Th2 (CXCR3-, CCR4+ and CCR6-), Th17 (CXCR3-, CCR4+ and CCR6+), Th1-17 (CXCR3+, CCR4- and CCR6+), Th1-2 (CXCR3+, CCR4+ and CCR6-), Th1* (CXCR3+, CCR4+ and CCR6+), or CCR Neg (CXCR3-, CCR4- and CCR6-) lineages (21–23). For PTM GAD specific cells, we observed that Th1-like cells were predominant, followed by Th1-2, Th1-17, Th1*, Th2, and Th17; a notable proportion were chemokine receptor negative and a minor proportion were positive for CCR6 only (**Figure 3C**). To juxtapose this chemokine receptor data with intracellular cytokine data, we combined all interferon-γ-associated (Th1, Th1-2, Th1-17, and Th1*) IL-4-associated (Th2 and Th1-2), and IL-17-associated (Th17, Th1-17, Th1*, and CCR6 only) lineage marker combinations (**Figure 3E**). Taking the average across all subjects with T1D, interferon-γ-associated surface marker expression were predominant, followed by IL-17-associated, and Th2-associated cells. This raised the possibility that the relative proportion of IL-17 producing cells may be underestimated in the cytokine data set. We performed the corresponding analysis for PTM IA2 specific cells, observing that Th1-like cells were predominant, followed by Th1-2, Th2, Th1*, Th1-17, and Th17; a notable proportion were chemokine receptor negative and a minor proportion were positive for CCR6 only (**Figure 3D**). Taking the average across all subjects with T1D, interferon-γ-associated cells were predominant followed by IL-4-associated, and IL-17-associated cells (**Figure 3F**).

### PTM specific T cells have distinct attributes in subjects with high or low residual c-peptide

**Figure 4:**
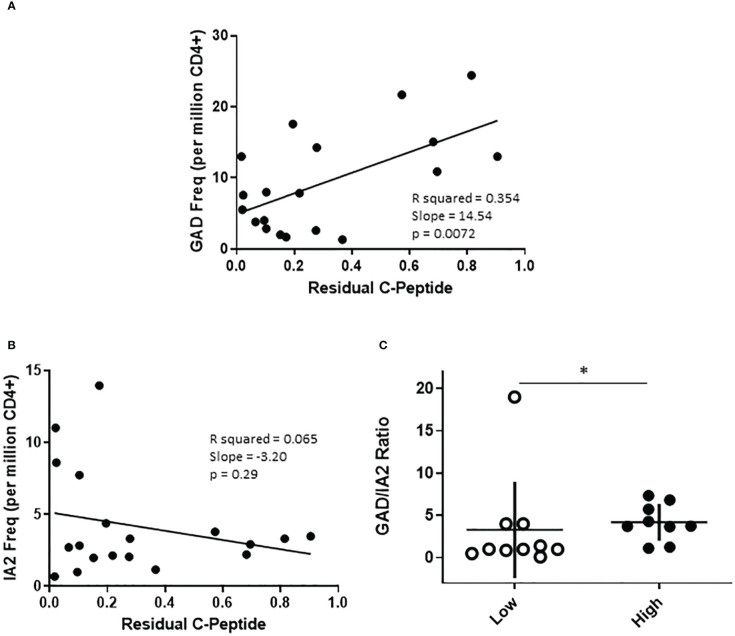
The profiles of PTM specific T cells are distinct in subjects with high or low residual c-peptide. The subjects with T1D in our study were recruited to have diverse levels of residual c-peptide. **(A)** Examining PTM GAD specific T cells revealed a linear relationship between the frequency of PTM GAD-specific T cells and the level of residual c-peptide (p=0.0072, simple regression). **(B)** However, there was not a significant linear correlation between the frequency of PTM IA-2-specific T cells and the level of residual c-peptide (p=0.29, simple regression). **(C)** Grouping subjects based on low (<0.2 mm/L, n=10 open circles) or high (>0.2 mm/L, n=9 filled circles) residual c-peptide revealed that the low group had a significantly lower proportion of PTM GAD-specific T cells (expressed as GAD/IA2 frequency ratio) than the high group (p=0.05, Mann Whitney test). Horizontal lines indicate the mean value for each group. Vertical lines indicate standard deviations.

The cohort of subjects with T1D recruited for this study were selected to have a wide range of residual beta cell function as measured by peak c-peptide in a mixed meal tolerance test. To probe relationships between T cell frequency and residual c-peptide, we performed linear regression analyses, considering residual peptide as a continuous variable. This revealed significant positive correlations between PTM GAD specific T cell frequency (p=0.0072, **Figure 4A**) and peak c-peptide; there was a lack of correlation between IA-2 specific T cell frequency and peak c-peptide (p=0.29, **Figure 4B**), perhaps in part because these frequencies were much lower. Our cohort included similar numbers of individuals with comparatively low (peak c-peptide < 0.2 nmol/L) or high (peak c-peptide > 0.2 nmol/L) residual beta cell function, but who were well matched with regard to disease duration, age at diagnosis, HbA1c, and autoantibody status (**Supplemental Figure 1**). Therefore, we also divided our cohort and compared antigen specific T cell frequencies between the high and low c-peptide groups. We observed no significant differences in total, PTM GAD, or PTM IA2 specific T cell frequencies between high and low c-peptide groups (**Supplemental Figure 5**), but there was a trend toward higher PTM GAD frequencies in subjects with T1D (p=0.16) and we noticed that elevated PTM IA2 frequencies were exclusively seen in the c-peptide low group. Reflective of those differences, the ratios of PTM GAD to PTM IA2 specific T cell frequencies were significantly lower in the c-peptide low group (p=0.05, **Figure 4C**).

### PTM specific T cells have distinct cytokine profiles in at-risk subjects

**Figure 5:**
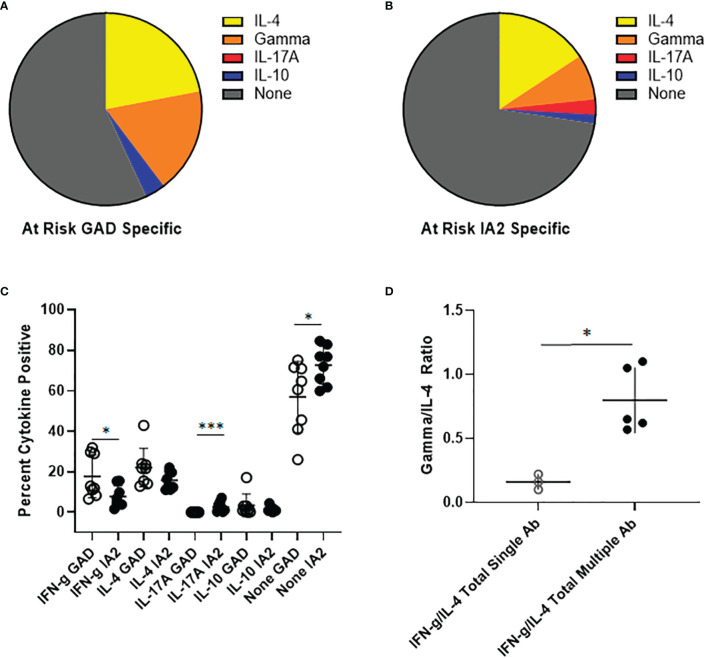
PTM specific T cells have distinct cytokine profiles in at-risk subjects. PTM GAD and IA-2 epitope specific T cells were characterized by intracellular cytokine staining after a single round of *in vitro* expansion. **(A)** The cytokine profiles of PTM GAD specific T cells were examined for 8 antibody positive at-risk subjects. Taking the average across all subjects, IL-4 was the predominant cytokine (22%) followed by interferon-γ (17.8%), IL-10 (3.4%), and IL-17A (0.03%); a notable proportion were cytokine negative (57%). No notable dual cytokine production was observed. **(B)** The cytokine profiles of PTM IA-2 specific T cells were examined in the same 8 at-risk subjects. Taking the average across all subjects, IL-4 was predominant (15.7%) followed by interferon-γ (7.6%), IL-17A (2.6%), and IL-10 (1.5%); a major proportion were cytokine negative (72.6%). Again, no notable dual cytokine production was observed. **(C)** Comparing PTM GAD (white circles) and IA-2 specific T cells (black circles) in at-risk subjects, a significantly higher percentage of PTM GAD specific T cells were interferon-γ positive (p=0.049, Mann Whitney test) and a significantly lower percentage were IL-17A positive (p=0.0002, Mann Whitney test) or cytokine negative (p=0.049, Mann Whitney test). **(D)** Grouping subjects based on single (open circles) or multiple (filled circles) autoantibodies revealed that the single autoantibody group had significantly lower ratios of interferon-γ to IL-4 than the multiple autoantibody group (p=0.036, Mann Whitney test). Horizontal lines in each panel indicate the mean value for each group. Vertical lines indicate standard deviations. * indicates p-value below 0.05 but above 0.01 ** indicates p-value below 0.01 but above 0.001 *** indicates p-value below 0.001 but above 0.0001.

To investigate the relevance of PTM GAD and IA2 specific T cells in the at-risk setting, we obtained cryopreserved samples from autoantibody positive at-risk subjects. The number of cells available from these pediatric subjects was not sufficient for direct tetramer staining and enrichment. Therefore, we performed cytokine profiling after a single round of *in vitro* expansion, just as we had done for subjects with established T1D. T cell expansion was successful for a total of 8 subjects. In contrast to subjects with established T1D, for PTM GAD specific T cells IL-4 was the predominant cytokine, followed by interferon-γ and comparatively low proportions IL-10 and IL-17A producing cells (**Figure 5A**). Similarly, for PTM IA2 specific T cells IL-4 was the predominant cytokine, followed by interferon-γ and comparatively low proportions IL-17A and IL-10 producing cells (**Figure 5B**). For these subjects, no notable dual cytokine production was observed. Detectable levels of cytokine were somewhat more robust for PTM GAD than for PTM IA2, with significantly higher levels of interferon-γ (p=0.049) and a lower proportion of cytokine negative cells (p=0.049), but a greater proportion of PTM IA2 specific cells were positive for IL-17A (p=0.0002) (**Figure 5C**). Since subjects with more than one biochemically defined islet antibody are at a higher risk of progression we next probed for differences between at-risk subjects with single versus multiple autoantibodies. Perhaps reflective of the modest number of subjects per group, we observed no significant differences between the relative proportions of IL-4, interferon-γ, IL-17A, or IL-10 producing cells for either PTM GAD or PTM IA2 specific T cells (**Supplemental Figure 6**). However, subjects with multiple autoantibodies had significantly higher ratios of interferon-γ to IL-4 (p=0.036, **Figure 5D**) suggesting a more Th-1 polarized response in this higher risk group.

## Discussion

Prior work by our group and others supports a role for various classes of post-translationally modified and non-conventional neo-epitopes in T1D (8–10, 24, 25). Specifically, our prior work demonstrated that GAD65 and IA2 proteins are modified through the activity of tissue transglutaminase and peptidyl arginine deiminase enzymes, the activities of which are upregulated under conditions of inflammatory stress (10, 12). T cells that preferentially recognize such epitopes were present at elevated frequencies in subjects with T1D and showed some evidence of a Th1-like phenotype (10, 12). In the present study, we simultaneously characterized the frequency and phenotype of T cells that recognize enzymatically modified neo-epitopes from GAD and IA2 in subjects with established T1D and examined their cytokine profiles in subjects with type 1 diabetes and at-risk subjects. Our objective was to extend previous studies by directly comparing the relative number and phenotype of PTM GAD and PTM IA2 specific T cells and more broadly characterizing their surface phenotype and functional profiles. To gain a clearer understanding of their significance, we compared subgroups of participants who had high or low levels of residual c-peptide and examined functional responses to these epitopes in at-risk subjects.

Utilizing previously developed HLA class II tetramers, we detected T cells that recognize PTM GAD or PTM IA2 epitopes in the peripheral blood of subjects with established T1D. As has been noted in previous studies, T cell frequencies were diverse but we observed consistently higher combined frequencies (GAD plus IA2) and GAD frequencies in subjects with T1D versus controls. IA2 frequencies were not significantly elevated in subjects with T1D, but four subjects had frequencies above 6 cells per million, levels that were never observed in controls. A paired analysis revealed that GAD frequencies were significantly higher that IA2 frequencies; indeed only four subjects had higher observed IA2 frequencies (the four with the highest measured frequencies). The cohort of subjects with diabetes included in this study were well matched with regard to age at diagnosis, disease duration, age, HbA1c, and antibody profiles, but were selected to have diverse levels of residual beta cell function. Examining PTM T cell frequencies with regard to residual c-peptide, we observed a significant positive correlation with combined PTM GAD and IA2 frequencies. This may have been driven by PTM GAD frequencies, which also had a significant positive correlation with c-peptide. In contrast, PTM IA2 frequencies were not significantly correlated with c-peptide, but these exhibited a weak trend toward being negatively correlated and the four subjects with the highest measured frequencies each had low c-peptide levels (<0.2 nmol/L). Indeed, comparing subjects with low (<0.2 nmol/L) versus high (>.2 nmol/L) levels of c-peptide, the low group had a significantly lower proportion of PTM GAD-specific T cells (expressed as GAD/IA2 frequency ratio), indicating that PTM IA2 specific T cells are overrepresented in the low c-peptide group.

Analysis of the cytokine profiles of PTM GAD and IA2 specific T cells after a single round of expansion revealed that interferon-γ was the predominant cytokine for both antigen specificities. However, GAD specific T cells had more robust functional responses than IA2 specific T cells, including significantly higher proportions of interferon-γ and IL-4 positive cells and a significantly lower proportion of cytokine negative T cells. Compared with healthy controls, we observed significantly higher proportions of interferon-γ positive T cells in subjects with T1D, reinforcing the paradigm that Th1 polarized responses are pathogenic (26). With regard to surface phenotypes, a higher proportion of PTM IA2 specific cells had a conventional memory phenotype than PTM GAD specific cells (based on lack of on CD45RA expression). However, the proportion of memory cells was significantly higher in subjects with T1D (as compared with controls) only for GAD. Patterns of chemokine receptor surface expression on memory PTM GAD and IA2 cells were similarly diverse, with combinations corresponding with Th1, Th1-2, Th1-17, Th1*, Th2 each comprising more than 4% of tetramer labeled cells. Grouping these combinations into interferon-γ, IL-4, and IL-17 associated lineages, we observed a predominance of cells with interferon-γ-associated lineage marker combinations. This is in accord with our intracellular cytokine analysis. Taken together, these results indicate that PTM reactive T cells exhibit a range of cell surface marker combinations and functional phenotypes, but Th1-like memory phenotypes and interferon-γ secretion predominates in subjects with T1D. The observed differences in the relative number and memory differentiation of PTM GAD and IA2 could be interpreted to indicate that PTM IA2 specific T cells are mobilized earlier during disease progression (evidenced by a higher proportion of CD45RA negative cells), whereas GAD PTM specific T cells are maintained at higher numbers by persistent antigen (evidenced by higher frequencies of GAD specific T cells in subjects with higher levels of residual c-peptide).

Analysis of the cytokine profiles of PTM GAD and IA2 specific T cells in at-risk subjects revealed differences in their respective cytokine profiles. In agreement with our observations from subjects with established T1D, GAD specific T cells in at-risk subjects had more robust functional responses than IA2 specific T cells, including significantly higher proportions of interferon-γ and a significantly lower proportion of cytokine negative T cells. In the at-risk group, a significantly higher proportion of IA2 specific T cells were IL-17A positive. Stratifying our at-risk subjects based on number of positive autoantibodies, we observed that at-risk subjects with multiple autoantibodies had significantly higher interferon-γ/IL-4 ratios than those with single autoantibodies. These results raise the possibility that PTM reactive T cells may acquire an increased Th1-like polarization during disease progression.

Our study does have limitations. Unfortunately, the age at draw was significantly higher for available HLA matched healthy controls than for subjects with T1D. Therefore, some of the observed differences could be influenced by age. Although the phenotypic markers included in our direct tetramer-staining panel are more extensive than many prior studies, important lineages such as peripheral helper and stem cell-like T cells are not accounted for. Furthermore, although the direct comparison of PTM GAD and IA2 specific T cells is novel, our tetramer panel did not permit us to resolve individual peptide specificities or to assess responses to native GAD or IA2 epitopes. It could be informative to design future studies that include additional surface markers (such as PD-1 and CD95), to implement strategies to de-convolute T cell specificities more precisely, and to directly compare responses toward PTM and native GAD and IA2. Although we observed interesting differences between at-risk subjects with single or multiple autoantibodies, a relatively small study cohort was available and samples were cross sectional rather than longitudinal. Because of limited blood volumes, our assays were limited to cytokine profiling after a single round of expansion. Likewise, although our study was able to correlate variations in T cell frequency with levels of residual c-peptide, it may not have been adequately powered to detect more subtle differences in phenotype. Therefore, future studies designed to confirm our observations using longitudinal samples and to address these additional questions would be of clear value.

In all, our findings documented clear differences in the number and functional attributes of neo-epitope specific T cells based on source antigen, revealed potentially important alterations in the fine specificity of neoepitope-reactive T cells for subjects with different levels of residual beta cell function, and revealed a possible shift in the cytokine profiles of neoepitope-reactive T cells during disease progression. These findings further establish a pathogenic role for PTM-reactive T cells in T1D and support a paradigm in which the mobilization of T cell responses against discrete neo-antigens potentiates more rapid loss of beta cell function.

## Data availability statement

The original contributions presented in the study are included in the article/**Supplementary Material**. Further inquiries can be directed to the corresponding author.

## Ethics statement

The studies involving human participants were reviewed and approved by the Benaroya Research Institute (adult subjects) or Medical College of Wisconsin (pediatric subjects) Institutional Review Board. Written informed consent to participate in this study was provided by the participants and their legal guardian if under 18.

## Author contributions

HN performed cell sorting and tetramer assays, analyzed data and co-wrote the paper. DA-L performed intracellular cytokine staining and tetramer assays, analyzed data and edited the paper. IC produced HLA class II monomers and edited the paper. WK contributed important ideas and edited the paper. CS and CG assisted with subject selection, contributed important ideas, and edited the paper. MH assisted with subject selection, contributed important ideas, and edited the paper. EJ designed the research, summarized data, provided technical training to HN and DA-L and co-wrote the paper. All authors contributed to the article and approved the submitted version.
