# Supplementary material for: Characterizing T cell responses to enzymatically modified beta cell neo-epitopes
Source: Front Immunol. 2023 Jan 10;13:1015855. doi: 10.3389/fimmu.2022.1015855 (PMC9871889; doi:10.3389/fimmu.2022.1015855)
Supplement: Supplementary file 1 [file DataSheet_1.pdf]

Supplemental Table 1. T1D Subjects used for study

Subject ID[a]	Age[b]	Sex	Time Since Diagnosis[b]	Peak C-peptide (nmol/L)[c, d]	Detectable Auto-antibodies[e]	Assays Run
T1D #1	53	M	10 years	0.367	GAD	Tetramer, ICS
T1D #2	17	F	4 years	<0.017	IA2	Tetramer, ICS
T1D #3	40	F	4 years	0.904	IA2	Tetramer, ICS
T1D #4	52	F	8 years	0.682	GAD	Tetramer, ICS
T1D #5	16	M	4 years	0.275	GAD, ZnT8	Tetramer, ICS
T1D #6	18	F	6 years	0.218	IA2, GAD, ZnT8	Tetramer, ICS
T1D #7	21	F	4 years	0.096	IA2, GAD, ZnT8	Tetramer, ICS
T1D #8	29	F	5 years	0.066	IA2, GAD	Tetramer, ICS
T1D #9	26	M	3 years	0.172	IA2, GAD, ZnT8	Tetramer, ICS
T1D #10	23	F	6 years	0.023	IA2, ZnT8	Tetramer, ICS
T1D #11	34	F	4 years	0.02	IA2, GAD	Tetramer, ICS
T1D #12	39	F	12 years	0.103	GAD	Tetramer, ICS
T1D #13	38	M	3 years	0.103	IA2, GAD	Tetramer, ICS
T1D #14	47	F	5 years	0.695	IA2, GAD, ZnT8	Tetramer, ICS
T1D #15	35	M	3 years	0.152	GAD, ZnT8	Tetramer, ICS
T1D #16	48	M	5 years	0.195	GAD	Tetramer, ICS
T1D #17	26	F	7 years	0.278	(none)	Tetramer, ICS
T1D #18	27	M	5 years	0.814	GAD	Tetramer
T1D #19	27	F	3 years	0.573	GAD, ZnT8	Tetramer
T1D #20	45	F	4 years	NT	NT	Tetramer
T1D #21	23	F	4 years	NT	NT	Tetramer
T1D #22	41	M	7 years	NT	NT	Tetramer
T1D #23	19	M	5 years	NT	NT	Tetramer
T1D #24	49	M	6 years	NT	NT	Tetramer
T1D #25	51	M	6 years	NT	NT	Tetramer

[a]Subjects 1-19 were recruited through the T1D Exchange and subjects 20-25 were recruited through the Benaroya Research Institute Diabetes Registry

[b]The mean age of patients was 33.8 years. The mean time since diagnosis was 5.32 years.

[c]Peak c-peptide was measured through a mixed meal tolerance test in conjunction with study enrollment. The limit of detection for the assay is 0.017 nmol/L. Results below that limit are reported as <0.017.

[d]NT indicates that test results were not available

[e]GAD denotes glutamic acid decarboxylase 65, IA2 denotes tyrosine phosphatase-related islet antigen 2, ZnT8 denotes zinc transporter 8. Autoantibody measurements were done in conjunction with study enrollment, but did not include insulin (IAA).

Supplemental Table 2. Healthy subjects used for study

Subject ID	Age[a]	Sex	Assays Run
Control #1	57	F	Tetramer, ICS
Control #2	30	F	Tetramer, ICS
Control #3	54	F	Tetramer, ICS
Control #4	66	F	Tetramer, ICS
Control #5	29	M	Tetramer, ICS
Control #6	57	F	Tetramer, ICS
Control #7	37	M	Tetramer, ICS
Control #8	27	M	Tetramer, ICS
Control #9	63	F	Tetramer, ICS
Control #10	58	F	Tetramer, ICS
Control #11	32	M	Tetramer, ICS
Control #12	33	F	Tetramer, ICS
Control #13	44	M	Tetramer
Control #14	66	F	Tetramer
Control #15	28	M	Tetramer
Control #16	42	F	Tetramer

[a] The mean age of controls was 44.8 years

Supplemental Table 3. At risk subjects used for study

Subject ID	Age[a]	Sex	Detectable Auto-antibodies[b]
Aab+ #1	2.4	M	IA2
Aab+ #2	14.6	F	GAD
Aab+ #3	17.1	F	GAD
Aab+ #4	12.5	F	IA2, ZnT8
Aab+ #5	14.0	M	IA2, IAA
Aab+ #6	13.4	F	IA2, IAA, ZnT8
Aab+ #7	9.3	F	IA2, IAA, ZnT8
Aab+ #8	12.2	M	IAA, ZnT8

[a]The mean age of at risk subjects was 11.9 years.

[b]GAD denotes glutamic acid decarboxylase 65, IA2 denotes tyrosine phosphatase-related islet antigen 2, IAA denotes insulin, ZnT8 denotes zinc transporter 8. Autoantibody measurements were done in conjunction with study enrollment.

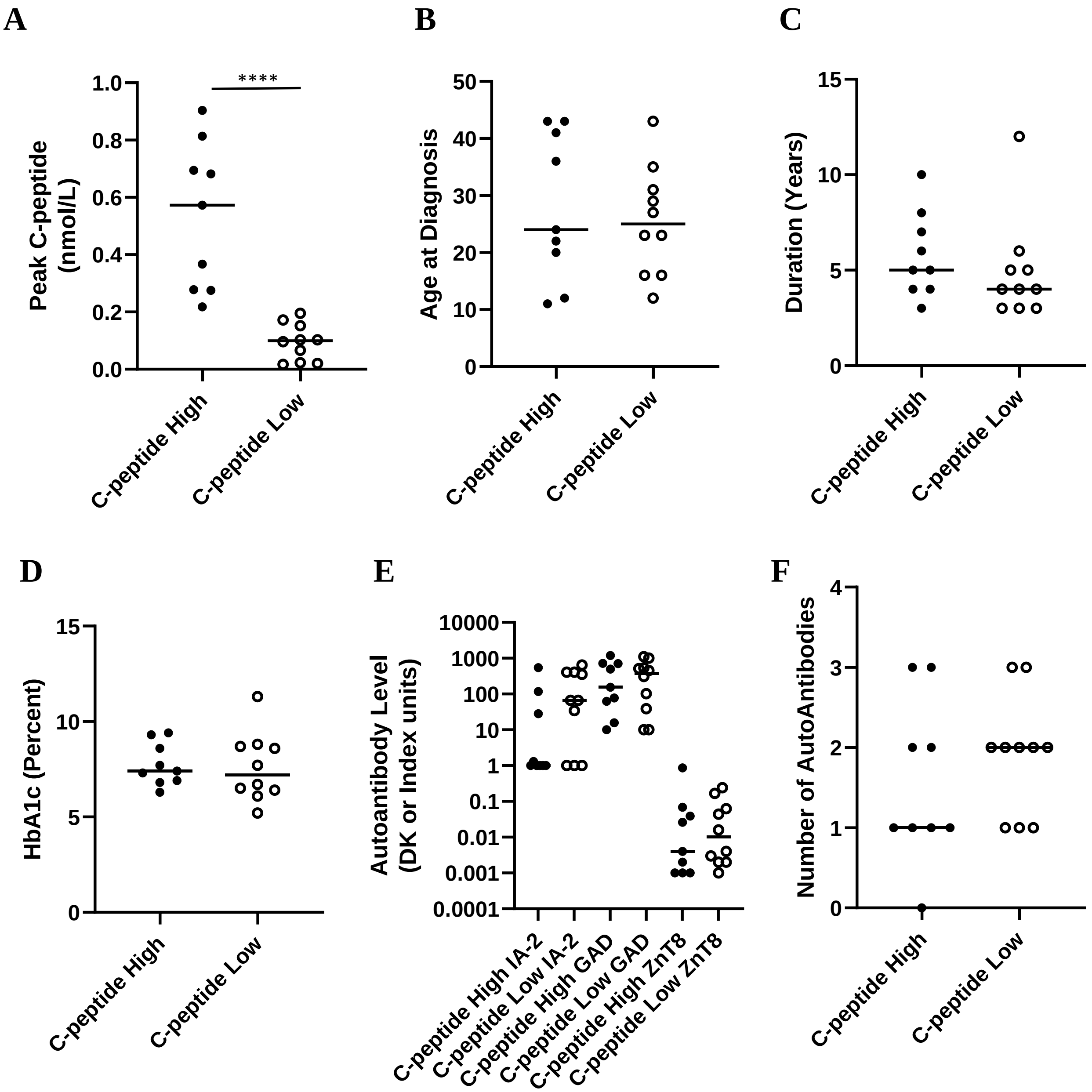

Supplementary Figure 1: Clinical attributes of subjects with T1D and high or low levels of residual c-peptide are well matched. Subjects with T1D were recruited for this study on the basis of having DR0401/DQ8 haplotypes and either low (≤ 0.1 nmol/L) or high (> 0.2 nmol/L) peak c-peptide at the time of enrollment. (A) By definition, the c-peptide high group (filled circles) had significantly higher levels of peak c-peptide (p<0.0001, Mann-Whitney) than the c-peptide low group (open circles). The two groups were not significantly different with regard to (B) age at diagnosis (p=0.628, Kolmogorov-Smirnov), (C) disease duration (p=0.889, Kolmogorov-Smirnov), (D) HbA1c (p=0.5901, Mann-Whitney), (E) level of IA-2, GAD, or ZnT8 antibody (p=0.172, 0.999, and 0.587, Mann-Whitney), or (F) number of positive autoantibody levels (p=0.650, 2x2 Chi-square; p=0.608, 2x3 Chi-square).

A

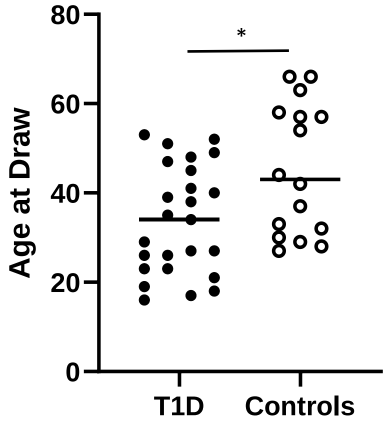

B

	Male	Female
T1D	11	14
Control	6	10

Supplementary Figure 2: Subjects with T1D and healthy controls are imperfectly matched. Subjects with T1D and healthy controls were recruited for this study on the basis of having DR0401/DQ8 haplotypes. The T1D and control groups were significantly different with regard to age at draw (p=0.0115, Mann-Whitney) but did not differ with respect to their sex distribution (p=0.753, 2x2 Chi-square).

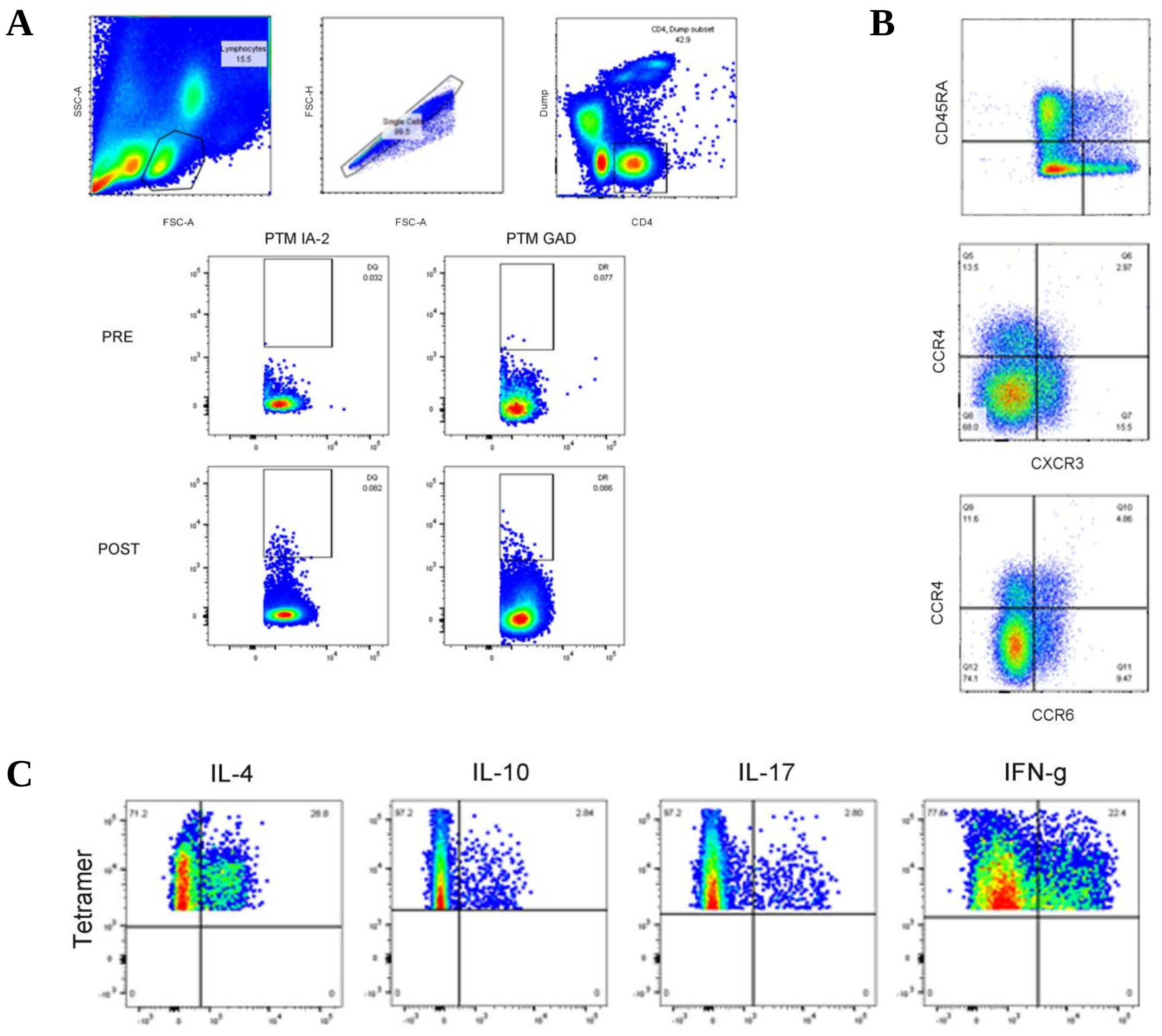

Supplemental Figure 3 – Surface marker gating for T cell analysis. FACS plots from a representative subject with T1D show the gating scheme used for CD4 T cell analysis. A: We sequentially gated tetramer and antibody stained peripheral blood as lymphocytes (FSC-A versus SSC-A), single cells (FSC-A versus FSC-H), and viable CD4+ T cells (CD4 versus Dump). For viable CD4+ T cells, positive thresholds were then set for each tetramer channel (PE-CF594 for PTM IA-2 and PE for PTM GAD) using pre-enriched cells (PRE) and applied to the magnetically enriched tetramer positive cells (POST). B: For viable CD4+ T cells, positive thresholds were also set to define memory T cells (CD45RA) and T cells positive for CXCR3, CCR4, and CCR6. C: Cultured primary T cells were similarly gated for viable, tetramer positive CD4+ T cells after fixation, permeabilization and staining with anti-cytokine antibodies. Positive thresholds were then set for each cytokine (as exemplified in each representative panel), including IL-4, IL-10, IL-17, and IFN-gamma.

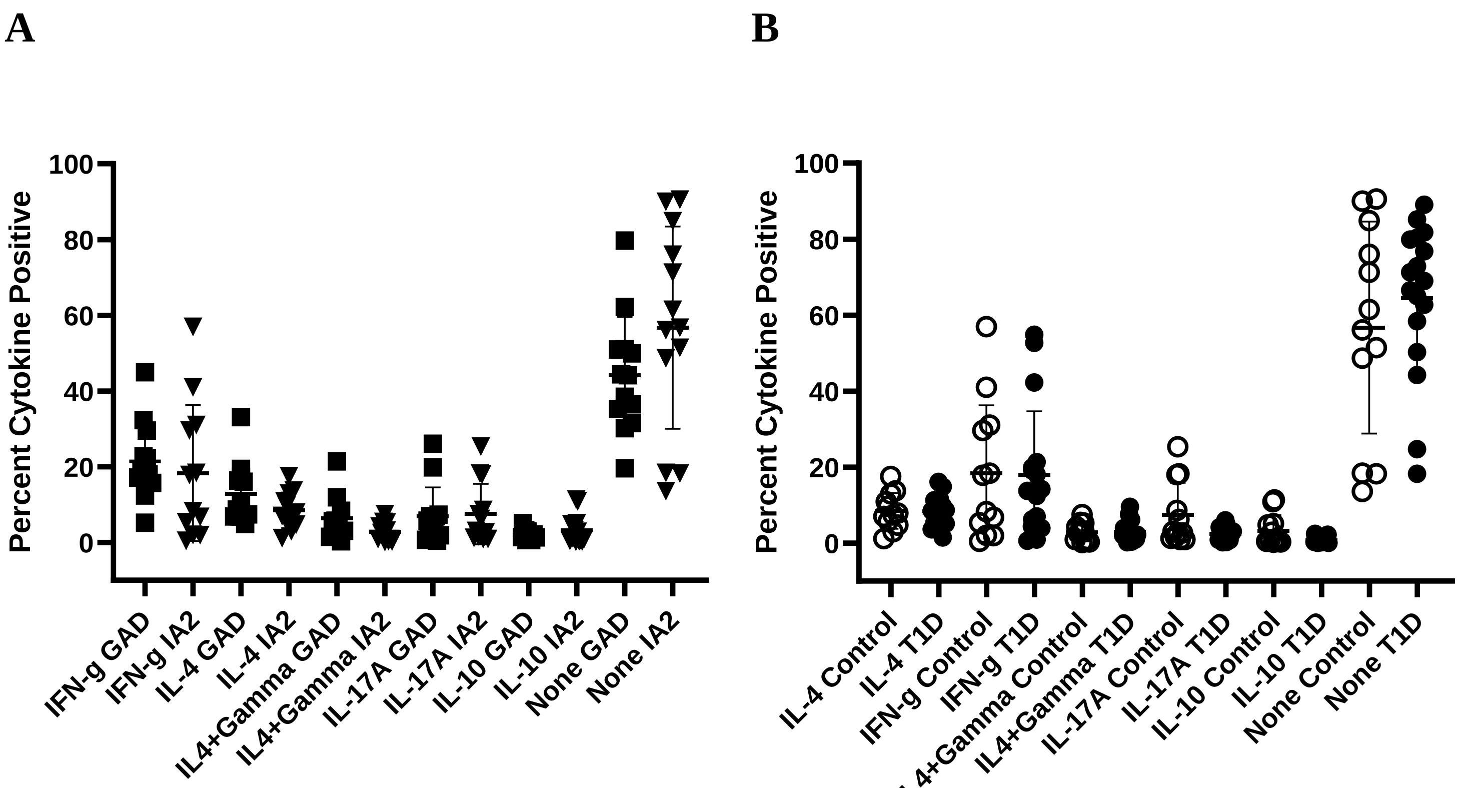

Supplementary Figure 4: PTM IA-2 epitope specific T cells have similar cytokine profile in subjects with T1D and controls. PTM GAD and IA-2 epitope specific T cells were characterized by intracellular cytokine staining after a single round of in vitro expansion. (A) Comparing PTM GAD (square symbols) and IA-2 specific T cells (inverted triangles) in control subjects, no differences in cytokine production were significant. (B) Comparing subjects with T1D (filled circles) and controls (open circles), there were no significant differences in the percentage of PTM IA-2 specific T cells that were cytokine positive.

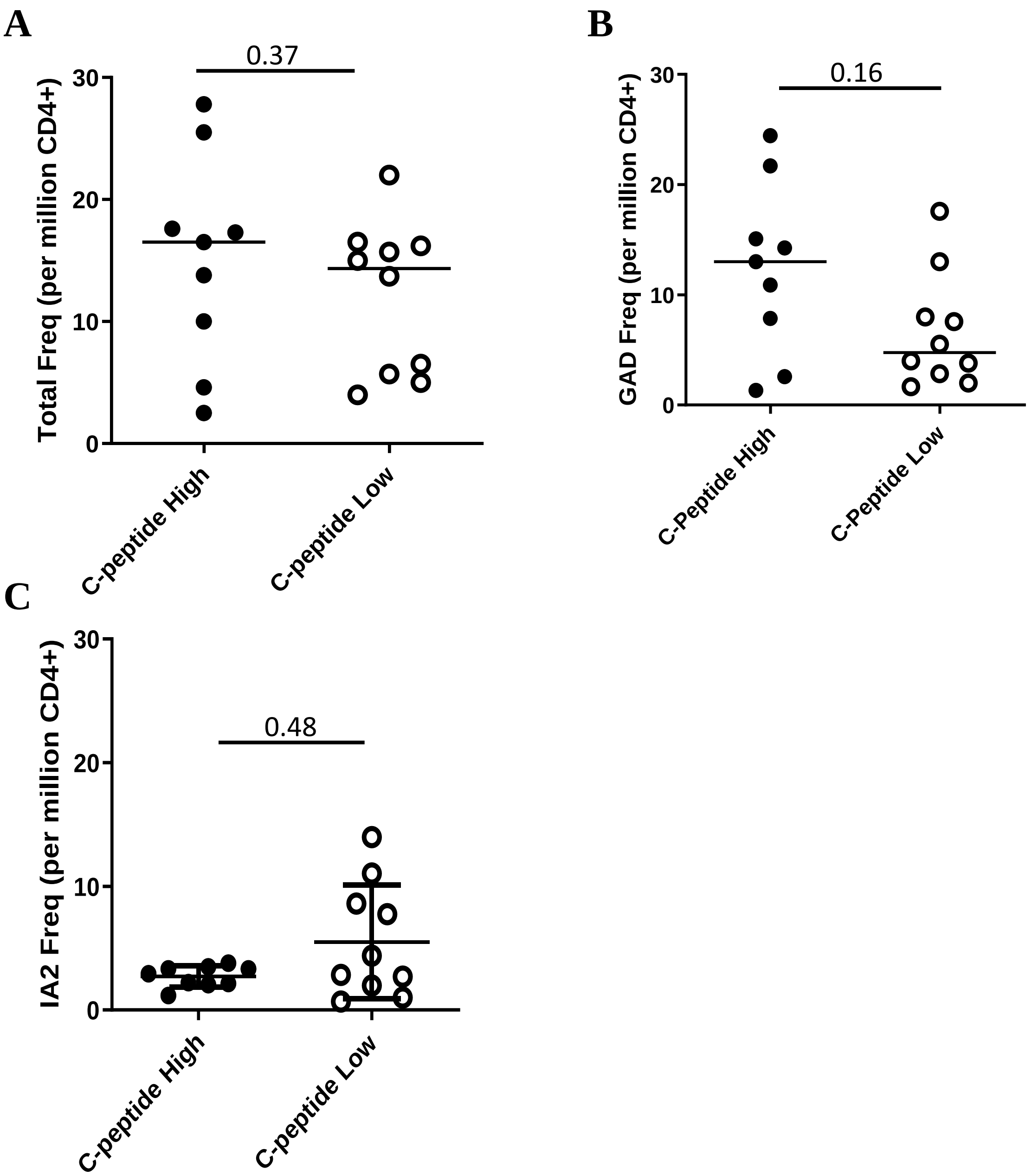

Supplementary Figure 5: PTM GAD and IA-2 epitope specific T cells have similar frequencies in subjects with high or low residual c-peptide. Subgroups of the subjects with T1D recruited for our study had either high (n=9) or low (n=10) levels of residual c-peptide. (A) Examining the combined frequency of GAD and IA2 specific T cells, there was no difference between subjects with high residual c-peptide (filled circles) and subjects with low residual c-peptide (open circles). (B) Examining modified GAD specific T cells separately, frequencies trended toward being higher in subjects with high residual c-peptide (filled circles) than in subjects with low residual c-peptide (open circles). (C) Examining modified IA2 specific T cells separately, frequencies were not significantly different between subjects with high residual c-peptide (filled circles) and subjects with low residual c-peptide (open circles).

A **B**

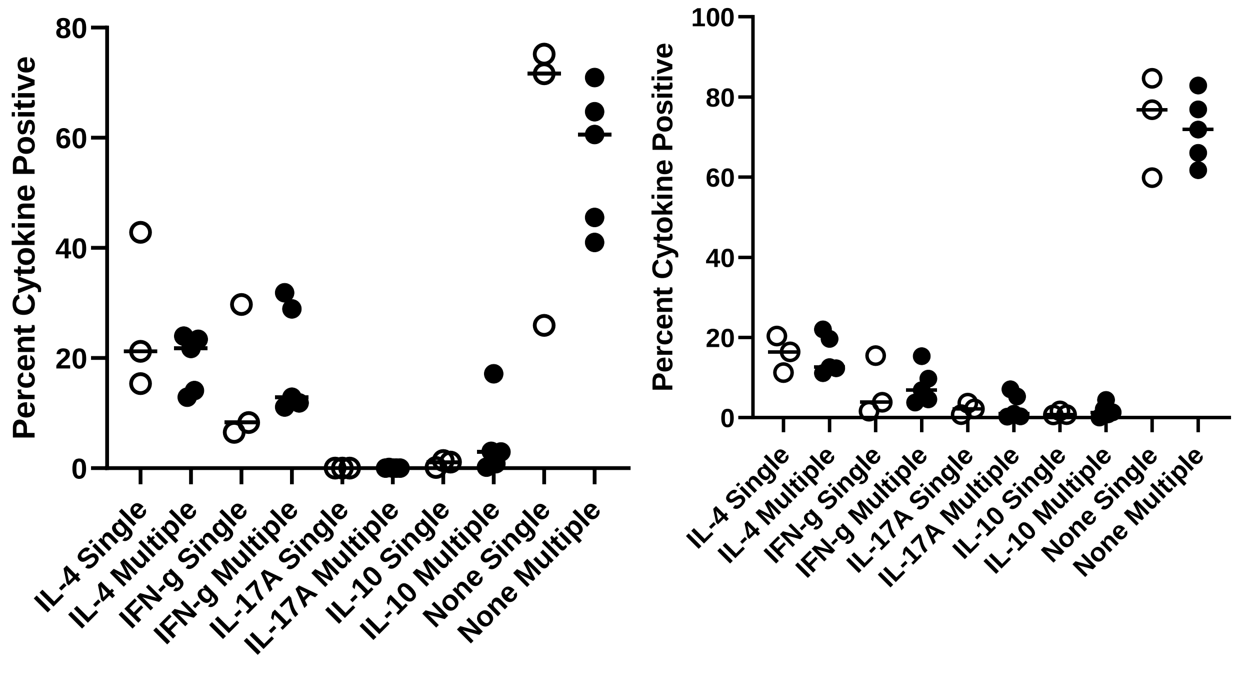

Supplementary Figure 6: PTM epitope specific T cells have similar cytokine profiles in at risk subjects. PTM GAD and IA-2 epitope specific T cells were characterized by intracellular cytokine staining after a single round of in vitro expansion. (A) Comparing at risk subjects with single (open circles) or multiple (filled circles) autoantibodies, there were no significant differences in the percentage of PTM GAD specific T cells that were cytokine positive or negative. (B) Likewise, there were no significant differences in the percentage of PTM IA-2 specific T cells that were cytokine positive or negative.
